# On Improving 5G Internet of Radio Light Security Based on LED Fingerprint Identification Method

**DOI:** 10.3390/s21041515

**Published:** 2021-02-22

**Authors:** Dayu Shi, Xun Zhang, Lina Shi, Andrei Vladimirescu, Wojciech Mazurczyk, Krzysztof Cabaj, Benjamin Meunier, Kareem Ali, John Cosmas, Yue Zhang

**Affiliations:** 1 Laboratory LISITE, Institut Supérieur D’électronique de Paris, 75006 Paris, France; dayu.shi@isep.fr (D.S.); lina.shi@isep.fr (L.S.); andrei.vladimirescu@isep.fr (A.V.); 2 Institute of Computer Scence, Warsaw University of Technology, 00-665 Warsaw, Poland; w.mazurczyk@tele.pw.edu.pl (W.M.); k.cabaj@elka.pw.edu.pl (K.C.); 3 Department of Electronic and Computer Engineering, Brunel University, Uxbridge UB8 3PN, UK; benjamin.meunier@brunel.ac.uk (B.M.); kareem.ali2@brunel.ac.uk (K.A.); john.cosmas@brunel.ac.uk (J.C.); 4 School of Engineering, University of Leicester, Leicester LE1 7RH, UK; yue.zhang@leicester.ac.uk

**Keywords:** visible light communication, LED fingerprint, 5G networks, security

## Abstract

In this paper, a novel device identification method is proposed to improve the security of Visible Light Communication (VLC) in 5G networks. This method extracts the fingerprints of Light-Emitting Diodes (LEDs) to identify the devices accessing the 5G network. The extraction and identification mechanisms have been investigated from the theoretical perspective as well as verified experimentally. Moreover, a demonstration in a practical indoor VLC-based 5G network has been carried out to evaluate the feasibility and accuracy of this approach. The fingerprints of four identical white LEDs were extracted successfully from the received 5G NR (New Radio) signals. To perform identification, four types of machine-learning-based classifiers were employed and the resulting accuracy was up to 97.1%.

## 1. Introduction

With the enormous growth of wireless devices and the deep integration of information technology into a number of industrial applications [1], the 5G network is expected to support massive user connections and exponentially increase wireless services. In addition, due to a tremendous number of Internet-of-Things (IoT) devices featured by the massive Machine-Type Communication (mMTC) extensive applications in the 5G network, high data rates, high connection density and ultra-reliable low latency communication (URLLC) should be provided. Moreover, security must be urgently assured by the future 5G network [2]. Hence, traditional radio frequency (RF) networks, which are already crowded, are arduous to satisfy these high demands [3]. One of the new communication technologies that has been proposed as an auspicious solution for the 5G and beyond is Visible Light Communication (VLC) [4]. VLC provides the nomadic access in hundreds of terahertz (THz) of unlicensed optical spectrum, immunity to electromagnetic interference, safety and security, simple implementation, and deployment of systems [5,6,7]. These exciting assets generate considerable research and industrial interests for the indoor VLC, especially with the approval of the IEEE 802.15.7 standard [8]. The research in this topic is conducted in the European H2020 project Internet of Radio Light (IoRL), which was the first to propose a hybrid indoor optical-radio network in convergence with the 5G Era [9,10,11]. The advantage of the VLC compared to radio [4] and its applications scenarios [12,13] has been widely discussed in the VLC literature. Due to the line-of-sight light propagation and the impermeability for non-transparent objects, the VLC channel exhibits higher security in a single-user or private-room scenario. However, in public areas such as classrooms, libraries, hallways, or planes, the security of the transmitted signal cannot be guaranteed [14].

Device fingerprinting, the process of gathering device information to generate device-specific signatures and using them to identify individual devices, has emerged as a potential solution for the 5G network to reduce the vulnerability of wireless networks to node forgery or insider attacks [15,16]. Its low-complexity and difficult or impossible to forge property could be perfectly matched with the security requirements of the 5G network. Notably, wireless device identification via Radio Frequency (RF) fingerprint becomes a widely concerned physical-layer security mechanism [17] and has been investigated in Wi-Fi, LTE (Long Term Evolution) and ZigBee systems. It provides an opportunity to accomplish the authentication and the target device identification in the physical layer. Recently, a RF fingerprint-based device identification method successfully demonstrated 92.29% identification accuracy on a set of seven 2.4 GHz commercial ZigBee devices [18]. It must be noted that device fingerprinting schemes can also be applied in the same spirit to VLC systems. However, the distinctive transmission protocols and modulation schemes of VLC systems such as intensity-modulation direct detection (IM/DD), direct-current (DC) biased optical orthogonal frequency-division multiplexing (DCO-OFDM) [19] necessitate the development of new device fingerprinting methods specific to VLC systems [20].

Currently, little research on VLC device identification exists. However, drawing on lessons from the successful implementations of device identification via RF fingerprint provides the possibility to propose the appropriate and efficient device identification method also for VLC systems. The concept of RF fingerprinting was firstly proposed by Hall et al. in 2003 [21]. This device identification method is featured by the detection of the transient signal emitted by the transmitter. Kennedy et al. was the first to introduce the device identification method based on the steady-state signal [22]. Afterwards, a large body of literature is dedicated to the general issues of design, implementation and identification algorithm relevant to many kinds of RF identification systems [23,24,25,26]. A comprehensive overview of high-level issues in the context of device identification method via RF fingerprint is summarized by Xu et al. [15].

That is why, in this paper, a device identification method is proposed based on the IoRL framework which is a typical indoor solution that integrates VLC-based communication, positioning and illumination systems within the 5G network [27,28]. This proposal aims to improve the security for the VLC-based 5G network under the public areas and broadcasting scenario as mentioned above. In this approach, fingerprints are extracted from the signal emitted by Light-Emitting Diodes (LEDs). These fingerprints are unique for every LED even if they are of the same type. Moreover, by employing a machine-learning-based classifier, the system is able to distinguish these fingerprints, thereby identifying with which device it is communicating. This fundamentally eliminates the threat of rouge base-station. It replaces the traditional encryption by a real-time and OTP (One Time Pad) encryption, which strengthens the overall security of the 5G networks. Moreover, the only prerequisites of this method are the received analog signal and some software modules, which means that this mechanism can be integrated into an existing 5G network physical layer. Considering the above, the main contributions of this paper can be summarized as follows:

- We introduce the LED fingerprint model based on the characteristics of the LED equivalent circuit and design the LED fingerprint extraction and identification mechanisms—by fitting the power spectrum. The parameters which represent the LED’s inherent and stable nature are chosen to constitute the LED feature vector. The feature vector of each LED forms its fingerprint. A multi-SVM (Support Vector Machine) classifier is investigated theoretically to illustrate the process of the fingerprint identification.
- We illustrate the conceptual design of a typical 5G VLC multi-access scenario using the proposed security solution. We present how LED fingerprinting could be used in real-life systems. To detail the process, we choose the IoRL project as an exemplary test system.
- We demonstrate the feasibility and accuracy of this method in a practical indoor VLC-based 5G network. During experimental evaluation, four identical LEDs were used to extract their fingerprints from the emitted 5G NR signals. Four machine-learning-based classifiers, i.e., decision tree, Naïve Bayes, SVM (Support Vector Machine), and KNN (K-Nearest-Neighbor) were employed to identify the extracted LED fingerprints. It turned out that the best results were achieved for the SVM classifier, which reached the accuracy of 97.1%.

The rest of this paper is organized as follows. Section 2 describes the proposed LED fingerprinting method in detail. Then, the experimental methodology and obtained results are detailed in Section 3. Finally, Section 4 concludes our work and outlines potential future directions.

## 2. LED Fingerprint Verification Mechanism

### 2.1. LED Fingerprint Model

**Figure 1:**
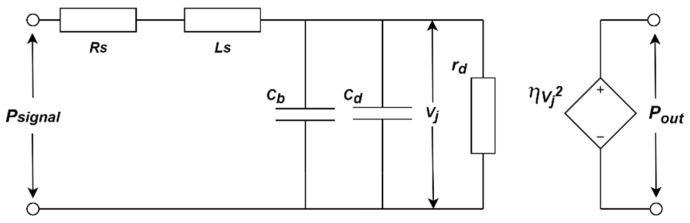
A Small-Signal Equivalent Circuit Model of an LED in the VLC System.

It is noteworthy that there is little difference between the components of a VLC system except for the LEDs. The nonlinearity and saturation characteristic support the possibility to distinguish LEDs, even those from the same batch [26]. Thus, the LED fingerprint model has been established to characterize the distinctive feature of each device. A small-signal equivalent circuit model of the LED in the VLC system is illustrated in Figure 1 According to the equivalent circuit, $p_{signal}$ is the power of the input signal, and $p_{out}$ is the power of the output optical signal. $R_{s}$ and $L_{s}$ are the parasitic resistance and parasitic inductance. $C_{d}$ and $C_{b}$ represent the diffusion and the barrier capacitance of the LED, respectively. *η* is the photoelectric conversion efficiency and $r_{d}$ is the small-signal diode resistance of an LED. The equations defining these elements using SPICE parameters are the following [29,30]:

(1) $$P_{optical}=\eta P_{electric}$$

(2) $$g_{d}=\frac{1}{r_{d}}=\frac{dI_{d}}{dV_{d}}=\frac{qI_{s}}{NkT}e^{qV_{D}/NkT}\approx \frac{I_{D}}{NV_{th}}$$

(3) $$C_{d}=TT\cdot g_{d}$$

(4) $$C_{b}=\{\begin{array}{cc} \frac{C_{J0}}{{\left(1-V_{D}/V_{B}\right)}^{M}} & V_{D}<FC\cdot V_{B}\\ \frac{C_{J0}}{{\left(1-FC\right)}^{1+M}}\left[1-FC\left(1+M\right)+\frac{MV_{D}}{V_{B}}\right] & V_{D}\geq FC\cdot V_{B} \end{array}$$

(5) $$C=C_{b}+C_{d}$$

(6) λ=[C,η]

The values of the diffusion capacitance $C_{d}$, the barrier capacitance $C_{b}$ and the photoelectric conversion efficiency η are the inherent and stable values of an LED. Once the fabrication of an LED is accomplished and the quiescent operation point was set, these values are fixed. Due to the industrialized and standardized production of LEDs, the dissimilarity of parasitic resistance and inductance values are too subtle to distinguish between different LEDs. Therefore, the values of η and C constitute a two-dimensional feature vector λ. It can represent the inherent characteristics of the LED and, as a result, we are able to define the feature vector of the LED and thus its fingerprint.

### 2.2. Extraction and Identification Mechanisms

**Figure 2:**
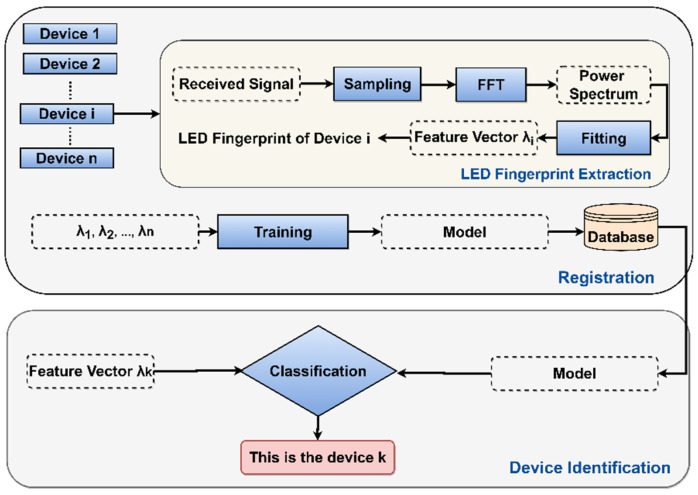
LED Fingerprint Extraction and Identification Mechanism.

In this section, introduced LED fingerprint extraction and identification will be explained. The extraction and identification mechanisms are illustrated in Figure 2.

The example of the LED fingerprint extraction of the *i*-th device will be described below in order to illustrate the proposed method’s functioning. The signal transmitted from the *i*-th device will be sampled by performing a Fast Fourier Transform (FFT) to calculate the power spectrum at the receiver. By fitting this measured power spectrum with its theoretical function, the feature vector becomes the LED’s fingerprint.

Before the identification, the registration of the authorized devices should be processed first. The feature vector of each authorized device will be extracted and put into a machine-learning-based classifier to train an identification model. Once the model has been established, it will be stored into a database which is integrated on the judgment module.

When a device *k* requests accessing, its feature vector *λk* will be extracted by the receiver. Meanwhile, the classification module will invoke the model stored in the database and perform identification. As the device has been recognized, the judgment module will decide if it permits it to access, based on the demands of the user.

### 2.3. Implementation of Features for Extraction and Identification

As described above, the signal transmitted by a device will pass through an optical wireless channel and be captured at the receiver. The power spectrum of this received signal will be fitted with its theoretical function to extract the feature vector as its fingerprint. The theoretical power spectrum function at the receiver $p_{Rx}\left(j\omega \right)$ is derived as follows:

(7) $$P_{Rx}\left(j\omega \right)=H_{LED}*H_{\mathrm{channel}}*G_{\mathrm{PD}}*G_{circuit}*P_{input}\left(j\omega \right)$$

(8) $$\omega =\left[2\pi f_{1},\;2\pi f_{2},\;2\pi f_{3},\ldots ,2\pi f_{n}\right]$$

(9) $$H_{LED}\left(j\omega \right)=\frac{\eta r_{d}Z_{in}}{{\left(\left(j\omega r_{d}C+1\right)\left(R_{s}+Z_{in}+j\omega L\right)+r_{d}\right)}^{2}}$$

(10) $$H_{channel}=\frac{Adet\left(m+1\right)cos^{m}\left(\phi \right)}{2\pi D^{2}}$$

where $H_{LED}$ is the power transfer function of the LED. It can be derived from the proposed LED equivalent circuit model. $H_{channel}$ is the power-loss function of the wireless optical channel [17]. $G_{PD}$ and $G_{circuit}$ are the power gain of the photodiode and the VLC front-end circuits, respectively. In this paper, the lights from the transmitter are considered to propagate only through line-of-sight (LOS), the non-line-of-sight (NLOS) propagation of lights is ignored. Based on the theoretical power spectrum function $P_{Rx}$ and the practical measurement $P_{rx}$, optimization can be employed to fit the feature vector *λ* as defined by (11):

(11) $$\underset{\lambda }{\min}\overset{2\pi f_{n}}{\underset{\omega =2\pi f_{1}}{\sum }}\left[P_{Rx}\left(j\omega \right)-P_{rx}\left(j\omega \right)\right]$$

Afterwards, all the feature vectors of devices from 1 to *n* will be extracted by the proposed method. The extraction result will be put into a model to train an identification classifier. Here, a multi-SVM classifier [31] is introduced as an example to solve the identification problem of M devices. We supposed that there are *N* training samples: $\left\{\lambda_{1},LED_{1}\right\},\ldots ,\left\{\lambda_{N},LED_{N}\right\}$. Here, $\lambda_{i}$ is the extracted fingerprint and $LED_{i}$ is the label that represents the feature vectors extracted from each LED. Subsequently, a one-against-all approach constructs M binary SVM classifiers, each of which separates one LED fingerprint from all the rest. Mathematically, ith SVM solves Equation (12) to yield the decision function (13):

(12) $$\{\begin{array}{c} \arg\;\min L\left(\omega ,\xi_{j}^{i}\right)=\frac{1}{2}{\left\|\omega_{i}\right\|}^{2}+C\sum_{l=1}^{N}\xi_{j}^{i}\\ y_{j}\left(\omega_{i}^{T}\phi \left(\lambda_{j}\right)+b_{i}\right)\geq 1-\xi_{j}^{i},\;\xi_{j}^{i}\geq 0 \end{array}$$

(13) $$f_{i}\left(\lambda \right)=\omega_{i}^{T}\phi \left(\lambda_{j}\right)+b_{i}$$

where $\xi_{j}^{i}$ denotes slack variables that are related to the soft margin, and *C* is the tuning parameter used to balance the margin and the training error. $\phi \left(\lambda_{j}\right)$ is the nonlinear mapping of $\lambda_{j}$. It maps the $\lambda_{j}$ to a much higher dimensional space in which the optimal hyperplane is found. During the classification phase, an unidentified feature vector λ is classified as $LED_{k}$ which decision function produces the largest value (14).

(14) $$f_{i}\left(\lambda \right)=\omega_{i}^{T}\phi k=\underset{i=1,\ldots ,M}{\arg\;\max}\;f_{i}\left(\lambda \right)=\underset{i=1,\ldots ,M}{\arg\;\max}\;\omega_{i}^{T}\phi \left(\lambda \right)+b_{i}\left(\lambda_{j}\right)+b_{i}$$

### 2.4. Envisioned Applications for the IoRL Security Framework

The threat that we would like to mitigate with the proposed LED fingerprinting method is as follows. The rogue device is a device placed by the attacker, which due to, e.g., a stronger signal, can lure users to connect to it and intercept valuable, confidential data send by the tricked users. Further in this paragraph we present how LED fingerprinting could be used in real-life systems. As an exemplary test system, we choose IoRL project [32]. The main subsystems of the IoRL system are optical RAN, UEs (User Equipment) and Intelligent Home IP Gateway (IHIPG). In the IHIPG, there is an OpenStack environment with SDN (Software Defined Network) which hosts various VNFs (Virtual Network Functions), for example, for security purposes. An example of the device that can be susceptible to such kind of attack is a LED device in the IoRL system. In effect, UE (User Equipment) receives data from the attacker’s LED, which, for example, could lead to installation of malware on it. Thus, the security system should detect such a situation and react accordingly. As already mentioned in the previous section, in this scenario we assume that during the registration phase before installation each new LED is tested in the laboratory environment. Its fingerprint is extracted to create a sort of “signature” of the LED for further use in the next phases and stored in the security system’s database enriched with the information where a given LED is physically installed.

Then, during the rogue LED detection phase, the security system periodically polls UE (using specialized software/firmware at UE) to establish the LED characteristics (i.e., LED fingerprint). The extracted LED fingerprints are sent to the security system for evaluation. Using these fingerprints and localization information security system verifies whether the extracted fingerprint is consistent with the fingerprint registered. In the case when the attacker placed a rogue LED device, the extracted fingerprint should be inconsistent with the fingerprint of the LED placed in the security system database during the registration phase. As a result, if the attack is detected, the security system can block the traffic directed towards/outgoing from the LED (so exfiltration of the confidential data obtained by the attacker is possible), and the administrators can be notified about this incident.

**Figure 3:**
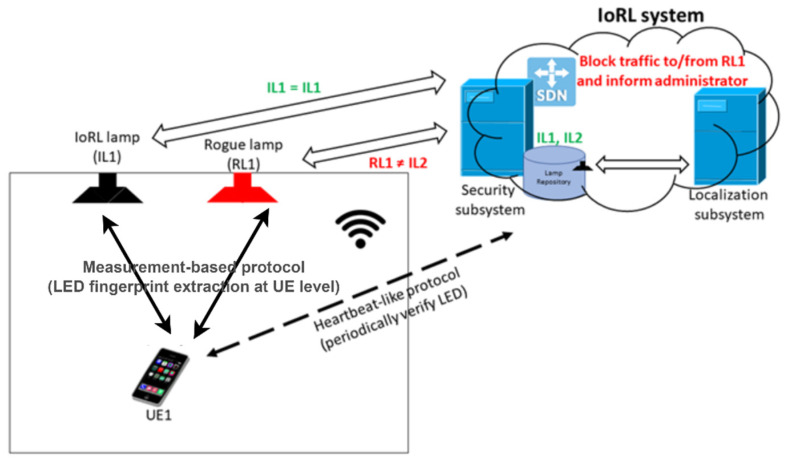
A block diagram of a typical 5G VLC multi-access scenario using the proposed security solution.

Figure 3 presents the main components used during the detection of rogue LED attacks. The main steps of the proposed detection mechanism are as follows:

- Step 0: Register LED fingerprint and their localization database (this information is established and inserted to the database before LED installation)—the database is located at the security system.
- Step 1: A heartbeat-like protocol (UE <-> security subsystem) with which the security subsystem would be able to periodically poll the UE to initiate the LED fingerprint extraction process and securely transmit the determined fingerprint back to the security subsystem.
- Step 2: The measurement-based protocol (UE <-> LED) that would operate between the selected UE and the LED which would allow to determine the fingerprint of the LED under investigation.
- Step 3: The mechanism at the security subsystem that would compare the fingerprint of the chosen LED and its location with the corresponding data stored in the database.
- Step 4: In the case of a detected security breach, the security system can install rules on the SDN controller to block incoming/outgoing traffic to the LED and notify the administrators.

## 3. Demonstration and Evaluation

In this section, the feasibility and accuracy of the proposed method was evaluated by a demonstration implemented in a practical indoor environment.

### 3.1. Demonstration Setup

**Figure 4:**
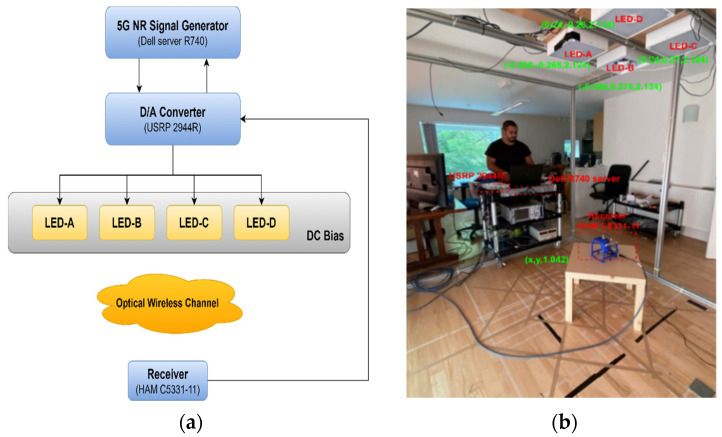

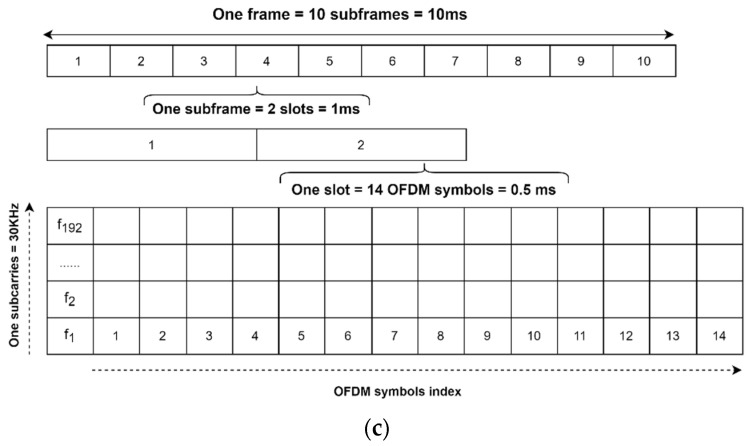
(**a**) A flow chart of the demonstration; (**b**) The realistic scenario; (**c**) The structure of 5G NR signal.

A flow chart of the demonstration and its realistic scenario are shown in Figure 4a,b. A server (Dell R740) acts as the 5G NR transmitter-generated 5G baseband signal. It modulates the user data with different modulation schemes which include QPSK, 16-QAM, 64-QAM and 256-QAM. As the modulated symbols were assigned to the 5G NR frame shown in Figure 4c, inverse Fast Fourier Transform (IFFT) processing and IQ modulation were applied to convert the OFDM frame to the time domain and guarantee the signal well matched with the IM/DD VLC system. Subsequently, a digital-analog (D/A) converter (USRP 2944R) and a DC bias transformed this digital signal to analog signal with an appropriate DC component. This signal is sent to four LEDs (LUXEN 5050). Through an optical wireless channel, the signal from each LED is captured by a commercial receiver (HAM C5331-11) in sequence and digitized by an analog-digital (A/D) converter (USRP 2944R). Finally, the received signal is transferred back to the server to extract fingerprints.

A 3D coordinate system was established to demonstrate the feasibility and accuracy of the proposed method for various distances and environments. According to Figure 4b, four LEDs were fixed on the ceiling in a known location. The receiver kept 1.042 m height and moved on a plane parallel to the ground. The distribution of the EVM (Error Vector Magnitude) of each LED in the whole area was tested to describe the optical wireless channel. Subsequently, signal samples from each LED were collected 10 times by the receiver at 24 different points. By using the proposed method, 960 LED fingerprints were extracted from all the samples and were randomly divided into training set and testing set in a ratio of 6:4. Several types of classifiers were employed to verify the accuracy of the extracted LED fingerprints.

### 3.2. Results and Analysis

**Figure 5:**
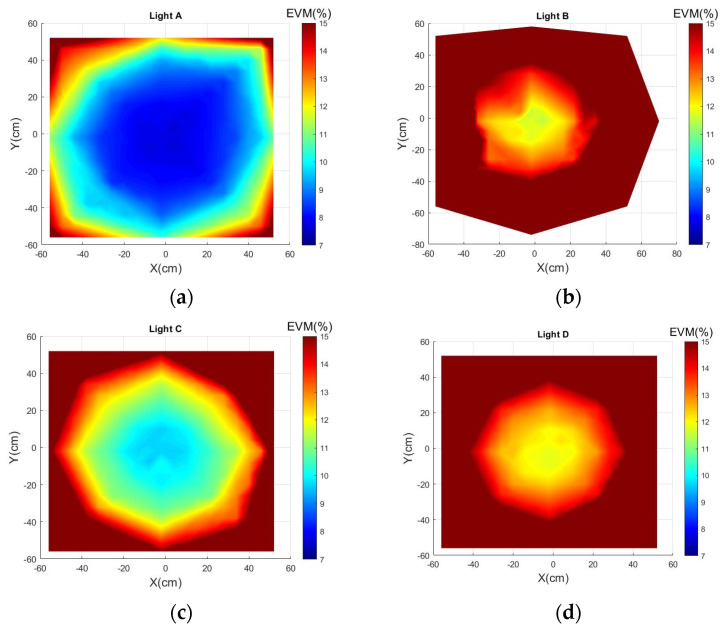
EVM distribution of LED in testbed: (**a**) EVM@LED-A; (**b**) EVM@LED-A; (**c**) EVM@LED-C; (**d**) EVM@LED-D.

Figure 5 illustrates 4 LEDs’ distribution of the EVM in the whole area. According to the results, LED-A and LED-C had the largest and the second-largest coverage of the high-quality communication environment. On the contrary, the channel of the LED-B and LED-D had limited quality. The phenomenon that the same type of four LEDs has different communication performance was caused by the non-linearity of the optical wireless channel.

**Figure 6:**
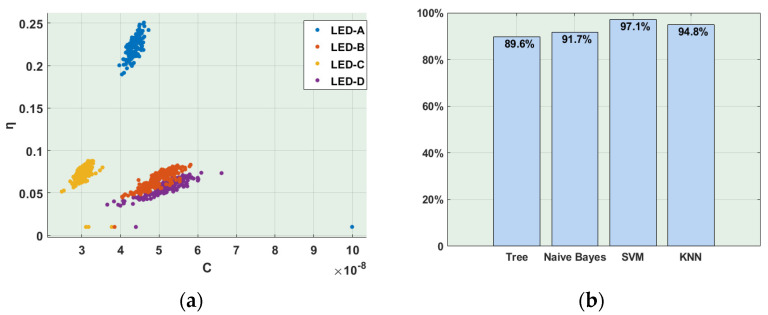
(**a**) Extracted LED fingerprints; (**b**) Comparison of verification accuracy.

Based on this environment, the total of 960 samples were captured by the receiver and, based on them, the LED fingerprints shown in Figure 6a were extracted. The extraction results were put into selected machine-learning algorithms, i.e., Decision Tree, Naive Bayes, SVM and KNN classifiers, respectively. The comparison of the verification accuracy is presented in Figure 6b. According to the obtained results, it is clearly visible that the proposed extraction method successfully extracted the LED fingerprints of four LEDs. Meanwhile, four machine-learning-based classifiers are able to distinguish these fingerprints appropriately.

**Figure 7:**
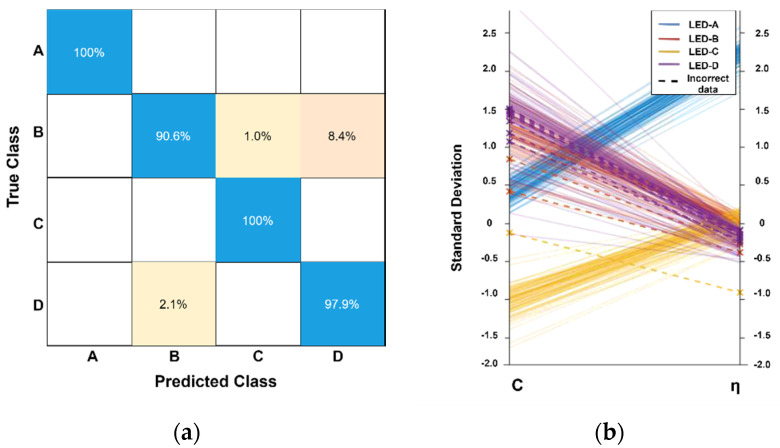
(**a**) The confusion matrix of the SVM classifier; (**b**) The parallel coordinate plots of the SVM classifier.

Note that further investigation leads to the conclusion that the fingerprints of the LED-B and LED-D overlapped in some regions, which was the primary reason of the classifiers’ misjudgment. In this case, the SVM classifier obtained the best performance due to its property of maximum classification margin and its ability of producing accurate and robust classification results for non-monotone and non-linearly separable input data. Moreover, the confusion matrix and parallel coordinate plots of the SVM classifier for the extraction and identification results are presented in Figure 7a,b.

According to the confusion matrix, almost all the misjudgment is caused by the LED-B as 8.4% LED fingerprints of LED-B were regarded as the fingerprints of the LED-D. Meanwhile, 2.1% fingerprints of the LED-D were misclassified as the fingerprints of the LED-B. Additionally, the parallel coordinate plots reflected the distinction of each parameter for all the fingerprints. The LED-A, LED-B and LED-C had distinct convergence on the parameter *C*. On the contrary, the LED-D performed more divergently and overlapped with the LED-B. For the parameter *η*, all the LEDs were convergent; however, the LED-B, LED-C and LED-D overlapped in a small interval. This phenomenon can be explained as follows. First, the identification quality is influenced by the communication environment. The LED-A has the best EVM and covered almost the whole area, which caused the highest identification accuracy. On the contrary, the LED-B yielded the worst accuracy due to its limited EVM coverage. Secondly, the parameter *η* is sensitive to the communication environment. According to the theoretical analysis of the extraction, *η* represents the photoelectric efficiency. Once there is a large attenuation in the channel, then the *η* will be calculated near the boundary value. Consequently, it will lose the ability to describe the characteristics of the LED.

## 4. Conclusions and Future Work

In this paper, a novel device identification method was proposed to improve the security of VLC in the 5G network. This method extracts the unique fingerprints of LEDs from their emitted signals. By employing a machine-learning-based classifier to distinguish these LED fingerprints, the devices accessing the 5G network can be reliably identified. The advantage of the proposed method is that it can work in real time and uses OTP (One Time Pad) encryption, which fundamentally avoids the threat of pseudo base-station and KI (Key identifier) disclosure. The feasibility and high accuracy of device identification were verified by using proof-of-concept implementation in a practical indoor VLC-based 5G network. In order to establish the optimal tuning of this approach, the comparison of four types of machine-learning-based classifiers was conducted. The obtained results indicate that the SVM classifier yields the best performance as it reaches the maximum accuracy of 97.1%. Additionally, the analysis of the confusion matrix and the parallel coordinate plots proved that the identification accuracy relies on the quality of the communication environment.

Our approach extracted the fingerprints of the transmitter in a VLC system to identify accessing devices. In future research, a bilateral identification mechanism which consists of Tx fingerprints and Rx fingerprints will be investigated to enrich the application scenario and further improve the security of the VLC system in the 5G network.

## Data Availability

Data sharing not applicable.
